# What Counts in the Immunological Synapse?

**DOI:** 10.1016/j.molcel.2014.04.001

**Published:** 2014-04-24

**Authors:** Michael L. Dustin

**Affiliations:** 1 Kennedy Institute of Rheumatology, Nuffield Department of Orthopedics, Rheumatology, and Musculoskeletal Sciences, The University of Oxford, Roosevelt Drive, Headington OX3 7FY, UK; 2 Skirball Institute of Biomolecular Medicine, Department of Pathology, New York University School of Medicine, 540 First Avenue, New York, NY 10016, USA

## Abstract

Molecular interactions at the interface between helper T cells and antigen-presenting B cells govern the ability to produce specific antibodies, which is a central event in protective immunity generated by natural infection or man-made vaccines. In order for a T cell to deliver effective help to a B cell and guide affinity maturation, it needs to provide feedback that is proportional to the amount of antigen the B cell collects with its surface antibody. This review focuses on mechanisms by which T and B cells manage to count the products of antigen capture and encourage B cells with the best receptors to dominate the response and make antibody-producing plasma cells. We discuss what is known about the proportionality of T cells responses to presented antigens and consider the mechanisms that B cells may use to keep count of positive feedback from T cells.

## Main Text

### Introduction

**Figure 1:**
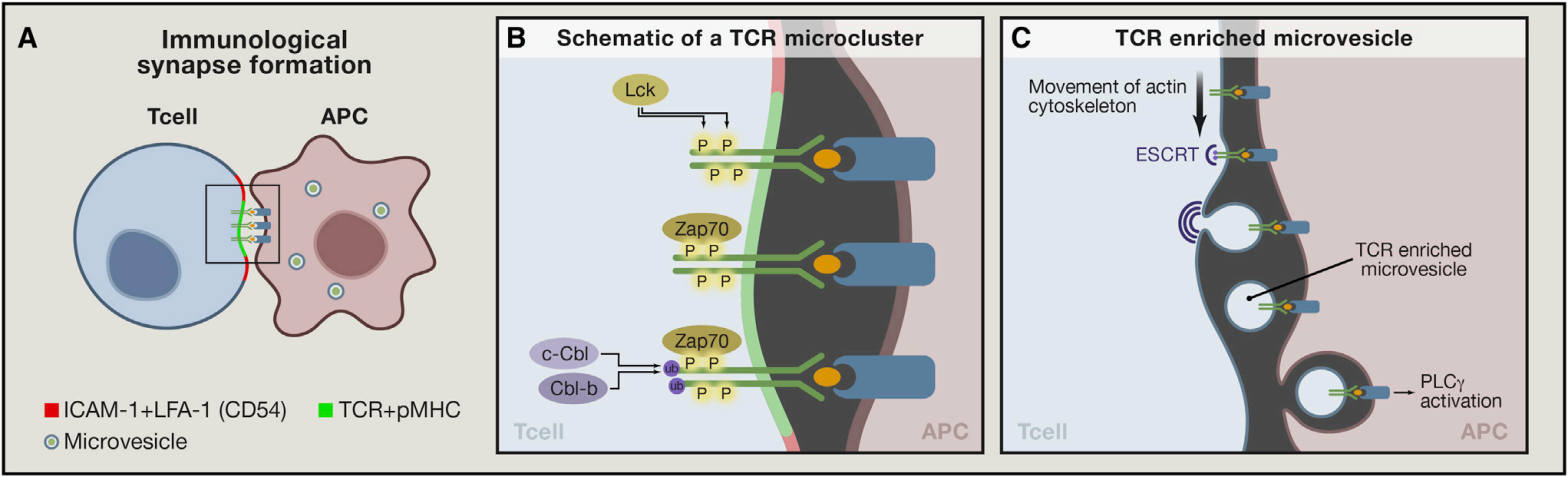
The Immunological Synapse, TCR Microclusters, and TCR-Enriched Microvesicles (A) Immunological synapse formation: when the T cell encounters the APC (antigen-presenting B cell) with appropriate MHC-peptide complexes, an immunological synapse forms with coarse segregation of TCR and bound peptide-MHC complex (pMHC) into the center (green) and a ring of LFA-1 (lymphocyte function-associated antigen 1) and ICAM-1 (intercellular adhesion molecule 1, a.k.a. CD54) (red). Microvesicles containing TCR-MHC-peptide interactions are generated from signaling microclusters, internalized by B cells, and induce signaling. The microvesicles are enriched in TCR, but their exact contents remain to be elucidated. (B) Schematic of a TCR microcluster: this is the site in which signaling is initiated. Following phosphorylation on tyrosine residues in the cytoplasmic domains of the TCR complex by Src family kinase Lck, the zeta-associated kinase of 70 kDa (ZAP-70) tyrosine kinase is recruited and assembles the TCR signalosome with substrates including Linker of Activate T cells (LAT) (Weiss and Littman, 1994). The TCR signalosome include ubiquitin ligases c-Cbl and Cbl-b, which add multiple mono-Ub to lysine’s residues of the TCR zeta chain (Naramura et al., 2002; Cormont et al., 2003). These are recognized by Tumor suppressor gene-101 (TSG-101) to initiate microvesicle formation once the microclusters reach a sorting domain just inside the integrin ring. (C) TCR-enriched microvesicles: optical-electron microscopy correlation has led to discovery of TCR enriched microvesicles. The actin cytoskeleton moves the microclusters downward in the schematic, and this also serves as a timeline for TCR microcluster and microvesicle formation. A signaling microcluster is initiated, the ESCRT machinery recognizes ubiquitin added to TCR in microclusters and sorts the TCR into plasma membrane buds that are released into the synapse center, and then the APC takes up the TCR-enriched vesicle, which can trigger PLCγ in the APC even in the absence of the T cell. This represents one of several mechanisms by which cells can transfer complex packets of information (Davis, 2007).

The production of high-affinity antibodies requires the formation of an immunological synapse between T and B cells. The synapse forms through the cooperation of two unique recognition systems: the T cell and B cell receptors, TCR and BCR (Victora and Nussenzweig, 2012). The bridges between these somatically diversified receptors are the products of the major histocompatibility complex (MHC), which incorporate small peptides derived from macromolecules captured and internalized by BCR and partly degraded in the B cell to form a composite ligand, referred to as the peptide-MHC complex, or pMHC. The pMHC is then recognized by the TCR in the immunological synapse (Lanzavecchia, 1985; Reinherz et al., 1999). Because the B cell utilizes its BCR to capture the antigen (Ag), or antibody-generating factor, the better the BCR affinity for the antigen, the more pMHCs are generated and recognized in the immunological synapse (Batista and Neuberger, 1998; Grakoui et al., 1999). The amount of pMHC generated by a B cell then becomes a surrogate for the quality of its Ag receptor and forms a basis for selection of B cells with the highest-affinity BCR to replicate, mutate, and differentiate into antibody-producing plasma cells. This framework is well agreed, but the details of how T cells discriminate different pMHC levels via the TCR and generate proportional feedback to B cells are not well understood. Recent studies suggest that the time that a TCR dwells with an individual pMHC (referred to as dwell time) in the synapse controls the T cell response. The helper T cell produces CD40 ligand (CD154) and cytokines for the B cells. But how CD154 is titrated by the T cell in response to pMHC dose and how the B cell remembers how much CD154 it has received through multiple cell divisions are not known (Hawkins et al., 2013). This review will focus the discussion on two key areas related to these challenges: how TCR discriminates pMHC quality and number at immunological synapses (Figure 1A), and potential mechanisms for how feedback can be provided to B cells that is proportional to pMHC.

### Can a T Cell Count?

The first wonder of the immune system is the ability of T and B cells to make antigen receptors by gene rearrangement, and the second wonder is the ability to make TCR ligands by peptide binding to MHC proteins (Babbitt et al., 1985; Bjorkman et al., 1987). The second process incorporates specialization of cytoplasmic (MHC class I) and endosomal (MHC class II) proteolytic machinery to generate the peptides and specific chaperoning of the respective MHC proteins to be receptive to peptide binding at the correct time and place to efficiently generate millions of these complexes on professional antigen-presenting cells (Trombetta and Mellman, 2005). Between the capriciousness of the proteases and some peptide binding preferences of specific MHC proteins, there can be large variability in how many antigens are needed to make one pMHC (Velazquez et al., 2001). However, it is reasonable to propose that the reproducibility of this process within B cells for a given pMHC, which is recognized by a clone of helper T cells, allows the immune system to use the number of pMHC generated as a surrogate for antigen uptake by the BCR (Batista and Neuberger, 2000; Fleire et al., 2006; Victora et al., 2010). If and how the helper T cell counts the pMHC and generates proportional feedback to the B cell is not known.

The problem of counting pMHC has been studied since the mid-1990s. Early experimental measurements of TCR-pMHC interactions in solution concluded that they were short-lived (Matsui et al., 1994). Valitutti and Lanzavecchia combined these observations with earlier observations about ligand-induced downregulation of TCR (Krangel, 1987) to formulate the serial triggering model (Valitutti et al., 1995). They made a simple assumption that productively engaged TCRs are lost from the surface over a period of minutes to hours. With this assumption, they measured TCR downregulation by flow cytometry, estimated the number of pMHC on APCs, and concluded that each pMHC must engage and lead to downregulation of 100 TCR. In this way, TCR downregulation appeared to be amplified by the presence of pMHC and provided a surrogate for T cell counting of pMHC. More recent data suggest that very high affinity or even covalent links of TCR to pMHC can result in efficient signaling (Xie et al., 2012). Therefore, the serial aspects may not be as important as the idea that the counting process is tied up with the fate of TCR after signaling. We will revisit this this point later. First, it is important to recognize that while serial triggering was a brilliant model that jumped way ahead of the technology to actually look at what was happening in an immunological synapse, the technology has caught up and made it possible to directly examine what happens between a TCR and pMHC in an immunological synapse. Therefore, in the context of this discussion of counting I will first discuss what we know about how the TCR interacts with pMHC in a model immunological synapse, as this relates to how the counting can take place.

### What Is Counted?

If the T cell could count, what would it count? The fundamental interaction that leads to triggering the TCR and could thus be counted is not certain. Physical measurements of many TCR-pMHC interactions have been made, and no measureable parameter in solution predicts biological outcomes across multiple TCRs. Short of direct measurements in synapses, there have been a number of efforts to generate predictive models based on solution interactions. Models that incorporate self pMHC into the triggering complex and that take into account rebinding of TCR and pMHC suggest the extreme high sensitivity and complex dependence of biological outcomes on the observable kinetics.

In the pseudodimer model, TCR forms a dimer based on binding one agonist pMHC class II, which also binds CD4, and one self-pMHC, which binds much more transiently. The TCR bound to the self pMHC is then efficiently phosphorylated by Lck (lymphocyte-specific protein tyrosine kinase) associated with the CD4 (Krogsgaard et al., 2005). This model was well supported by the ability of soluble heterodimers of agonist pMHC and self-pMHC to trigger T cell activation when applied in solution. However, O’Donoghue et al. demonstrated that single agonist pMHC would recruit up to six ZAP-70 kinases, which could be accommodated by the immunoreceptor tyrosine-based activation motif (ITAMs) of one TCR complex, calling into question the need for self-ligands to attain single-molecule sensitivity (O’Donoghue et al., 2013). A somewhat different effect of self pMHC is observed in CD8+ T cells (Yachi et al., 2007). In the case of CD8 T cells the supportive role of CD8-MHC class I interactions relates to adhesive avidity, as the bound peptide doesn’t matter. The effects of self-pMHC warrant further study, but as it is not clear that these are required to understand the counting conundrum, we will not discuss them further here.

In contrast to serial triggering, other investigators have considered the rebinding of the same TCR and pMHC in the interface to generate a new kinetic parameter, the dwell time (Aleksic et al., 2010; Govern et al., 2010). Dwell time is based on the concept of an encounter complex in which TCR and pMHC come together in an interface and are close enough to bind. Because diffusion in membranes is slower than in solution, formation of the encounter complex is rate limiting for binding, and once formed the slow diffusion may allow many cycles of binding and dissociation before the TCR and pMHC escape this encounter complex, particularly if other molecular systems are stabilizing the cell-cell junction and may confine diffusion of the TCR and pMHC. The theory behind these calculations was worked out in the 1970s (Bell, 1978), and support for rebinding in situ came from direct kinetic measurements in adhesive interfaces (Tolentino et al., 2008). The dwell time incorporates the kinetic on and off rates that can be measured in solution with information about diffusion of TCR and pMHC in the membrane (and eventually orientation factors) to determine the average number of times that a TCR and pMHC will rebind before diffusing apart—how long it stays in an encounter complex. Calculations of dwell time have been more successful in fitting solution-binding data to biological outcomes, and this is the current state of the art that has been invoked to explain complex decision making in T cell differentiation (Tubo et al., 2013). It is likely that such models can improve predictions by being internally consistent, without actually being physically accurate. So direct measurements could further improve predictions and generate new insights.

### Watching T Cells Count

The observation that T cells respond to single pMHC makes the T cell counting process in the synapse the perfect subject for single-molecule imaging. Models based on supported lipid bilayers (SLBs) and total internal reflection fluorescence microscopy are the perfect setting for single fluorophore imaging and even fluorescence resonance energy transfer at the single-molecule level. Huppa et al. and O’Donoghue et al. both performed single-molecule imaging in interfaces between live T cells and SLB with adhesion ligands and single pMHC (Huppa et al., 2010; O’Donoghue et al., 2013). Single-molecule tracking depends upon having systems where the ligand can be diluted to ∼0.1/μm^2^, a density which allows T cell activation in this model. These studies generate complementary information based on the use of rapidly evolving technology.

Huppa et al. (2010) used single-molecule FRET to determine the distance between the H57 Fab bound to the TCR and the C terminus of the peptide bound to the MHC in established immunological synapses. These are 4.1 nm apart in the crystal structure determined for H57 bound to the TCR and docked to the relevant pMHC (Wang et al., 1998). They found that the TCR-pMHC interactions spend only a short time, one-tenth the solution half-life, in this native configuration. This half-life of this conformation was increased to the full solution half-life by depolymerizing actin. This finding particularly supported models in which mechanical forces have been invoked in TCR triggering as the accelerated dissociation is a prediction of mechanical force acting on noncovalent interactions (Kim et al., 2009). While Huppa et al. (2010) interpreted their results in terms of an accelerated dissociation due to force, it has been suggested that force-dependent distortion of the complex without dissociation of the interaction could also account for the loss of FRET, as the process is very sensitive to distance. A limitation of the Huppa et al. (2010) study is that each pMHC could only be followed for about five frames, and thus the apparent off rates were a product of statistical analysis of brief observations on many different TCR-pMHC interactions, rather than following individual pMHC over longer times.

O’Donoghue et al. (2013) use much more photostable fluorophore to label the pMHC and thus could extend their measurements into the time frame of immunological synapse formation. Rather than use FRET to look at interactions, they examined the dramatic slowing of pMHC diffusion upon interaction with the TCR in the T cell-SLB synapse. This doesn’t measure the binding event in the way Huppa did but is actually a great way to measure a local dwell time. O’Donoghue et al. found a perfect correlation between the apparent off rate in the synapse and the solution off rate for several TCR-pMHC complexes and concluded that the interaction in the T cell-SLB system is very similar to solution. One caveat of comparing these measurements to those in cell-cell systems is that the diffusion coefficient for pMHC in the bilayer system is 10- to 100-fold higher than for a cellular system, and this could make rebinding more likely in the cell-cell system.

Huppa et al. (2010) and O’Donoghue et al. (2013) were both heroic data sets that have generated a wealth of information. Either the forces exerted in the synapse accelerate dissociation but allow for rebinding to coincidentally have a dwell time similar to the solution half-life, or the interactions are distorted by force but hold on to persist over a period identical to the solution off rate, after which the TCR and pMHC diffuse apart. Thus far, these measurements have only been made for one receptor system in which the solution half-life agreed quite well with the biological potency. We now need more heroic measurements with TCR and pMHC that do not show this correlation to determine if rebinding or different effects of cytoskeletal force are observed. It may also be necessary to slow the diffusion of the pMHC and to design additional FRET probes to distinguish conformational distortion from dissociation. Thus far, direct measurements in this model system that have directly demonstrated single-molecule sensitivity seem to show that the capture of the pMHC is both highly efficient and then persists for a period that is not dramatically different than the solution half-life. Events could be counted over time, but who is keeping track?

### B Cell Programming by Helper Cell Signals

The function of T cell help for B cells can be partly replaced by engaging CD40 and various cytokine receptors including IL-4 and IL-21. It has been noted that B cell responses to CD40L are graded, with division time and number of divisions being proportional to anti-CD40 dose in vitro (Hawkins et al., 2013). T cells contain preformed CD40L in secretory lysosomes (Koguchi et al., 2012) and focus this in the immunological synapse center (Boisvert et al., 2004), although whether this release process can be matched to pMHC dose or would have a threshold controlling a binary response is not known. In T cells, the signaling through the TCR appears to control future programing through duration of signaling above a threshold and the strength and nature of the signal (Iezzi et al., 1998). How signals from CD40 are integrated to control future cell cycles and differentiation in B cells is not known.

### Something Else for B Cells to Count

The serial triggering model emphasized TCR downregulation as a surrogate or engagement (Valitutti et al., 1995). The mechanism of downregulation is well studied for receptor tyrosine kinases like the epidermal growth factor receptor and is based on ubiquitination, internalization, sorting into multivesicular bodies by the endosomal sorting complexes required for transport (ESCRT), and degradation in lysosomes (Soubeyran et al., 2002). It was thought that the TCR faced a similar fate and all evidence supported this (Valitutti et al., 1997). Surprisingly, we determined earlier that the ubiquitination complex associated with T cell receptor downregulation was also important for normal formation of the immunological synapse (Dustin et al., 1998; Lee et al., 2003). TCRs are ubiquitinated, and this most likely takes place in microclusters in conjunction with the tyrosine kinase cascade (Figure 1B). We later established this was directly related to a role of ESCRT I in formation of the central TCR cluster in the immunological synapse in the T cell-SLB model (Vardhana et al., 2010). ESCRT I complexes directly recognize monoubiquitinated receptors and sort them into vesicles that bud into the lumen of the endosome, giving rise to what are called multivesicular bodies. The center of the immunological synapse contains many cellular organelles, so it was impossible to sort out the topology of TCR in this region by light microscopy. Optical-electron microscopy correlation studies on in CD4+ helper T cells revealed that this central, TSG101-dependent region of the immunological synapse contained arrays of many ∼60–100 nm vesicles that were highly enriched in TCR (Choudhuri et al., 2014). Use of a dominant-negative VPS4, a terminal ESCRT component that uses ATP to recycle ESCRT components and allow scission of the bud neck, results in TCR-enriched plasma membrane buds (Choudhuri et al., 2014). These results strongly suggest an alternative mode of TCR downregulation—the ESCRT-dependent budding of ubiquitinated TCR into the immunological synapse (Figure 1C). Analysis of T cell-B cell conjugates revealed bidirectional membrane transfer at the immunological synapse, with most T cells taking some pMHC from the B cell and most B cells reciprocally taking TCR from the T cell. The T to B cell transfer of TCR was blocked by knocking down ESCRT I. Thus, the TCR microvesicles are also generated in cell-cell immunological synapses and appear to be endocytosed by B cells from the immunological synapse. In addition, B cells demonstrated recruitment of phospholipase C-γ to the sites of intracellular TCR and fluxed Ca^2+^ due to contact with the TCR enriched microclusters in the absence of T cells, but they needed to express agonist pMHC for the TCR (Choudhuri et al., 2014). These studies suggest that after completing signaling in the T cell, the TCR becomes a ligand that facilitates the activation of B cells, and potentially other APCs.

**Figure 2:**
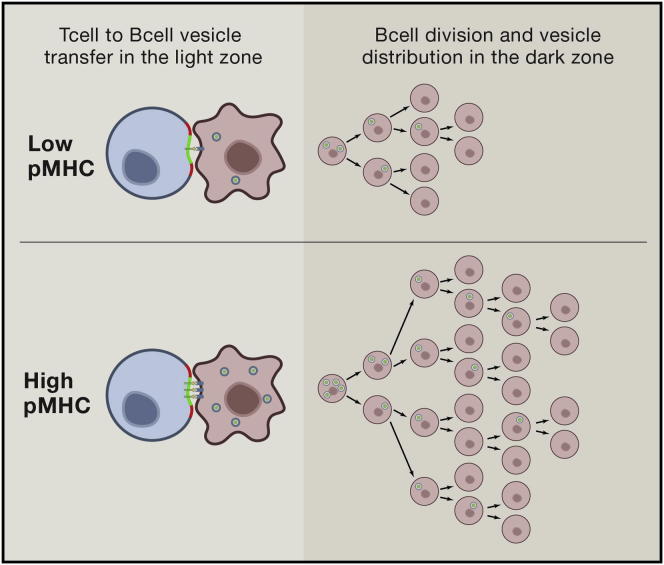
Microvesicle Programing of B Cells Model for role of TCR-enriched microvesicles in programing B cells for pMHC-linked cell division. High pMHC (affinity or quantity) leads to more TCR-enriched microvesicle transfer in the germinal center light zone. As B cells divide, they symmetrically segregate the TCR-enriched microvesicles and stop dividing when these are lost by division or consumed. This could be a basis for competition between high- and low-affinity B cells in germinal centers. The availability of microvesicles in the germinal center system would control the growth of the structures. The signals in the microvesicles may also include CD40L based on work with related exosomes and central localization of CD40 in the immunological synapse (Blanchard et al., 2002; Boisvert et al., 2004).

The production of TCR-enriched microvesicles is linear with pMHC density, making it one of the few outputs from T cells showing this strict relationship (Choudhuri et al., 2014; Grakoui et al., 1999). The linearity in this process with pMHC input has been consistently observed in the SLB-based system with different TCR, but it should be kept in mind that ways to document this in cell-cell systems or in vivo need to be developed. The details of this relationship are also complex as both the number of vesicles and the density of pMHC in the vesicles increases, such that there are two components to this effect that may not be biologically equivalent (Choudhuri et al., 2014). Nonetheless, it is interesting to think about how this initial indication of linearity of TCR-enriched microvesicle formation with pMHC might be biologically useful. Since B cells’ production of pMHC is linearly related to antigen uptake by their surface immunoglobulin (sIg) (Batista et al., 2001) and T cell help appears to control competition between different B cell clones in germinal centers (Victora et al., 2010), the TCR-enriched microvesicle accumulation in the B cells could act as a ligand depot that could continue to drive proliferation of the B cell as it separates from T cells. The germinal center, where antibodies undergo affinity maturation and class switching under the direction of helper T cells, has two zones—the light zone (LZ), where the T and B cells interact, and the dark zone (DZ), where the B cells leave the T cells and undergo division, class switching, and antibody mutation (Figure 2). B cells may accumulate TCR-enriched microvesicles in the LZ and then move to the DZ, where they divide until the TCR-enriched microvesicles are diluted to a point at which they no longer drive B cell activation. This model would predict that the more pMHC a B cell has, the more TCR-enriched microvesicles it can accumulate and the more it will divide. This process of generating membrane buds at the plasma membrane is very similar to viral budding, an in fact HIV appears to be able to directly hijack this pathway for polarized release of viral particles (Choudhuri et al., 2014). The particularly remarkable aspect is that T cell help for B cells may take advantage of a virus-like strategy to imprint B cells with a cell division program. The nature of the instruction is not known, and it seems unlikely that pMHC is the only receptor. CD40 ligation is a key signal delivered from helper T cells to B cells in germinal center, and including CD40L, a membrane protein, in microvesicles could provide a depot of this ligand.

The generation of TCR-enriched microvesicles could leave the counting to the B cells, which could potentially do this through a dilution-based mechanism that would not require the ability or either the T cell or the B cell to possess signal integration mechanisms with a large dynamic range. This would allow all the individual signaling processes to have switch-like characteristics, while generating a quantal unit in numbers that are roughly consistent with number of cell divisions undergone in the germinal center.

### Asymmetric Division Could Skew the Count

There are two types of asymmetric cell division that have been proposed to impact cell fate in the immune system—asymmetric division triggered by external niches such as the immunological synapse (Chang et al., 2007; Dustin and Chan, 2000), or asymmetric division based on partitioning of a unitary cytoplasmic organelle that can only be inherited by one cells and give that cell special characteristics (Thaunat et al., 2012). Thaunat et al. (2012) demonstrated that B cells inherit the antigen-laden compartment that forms pMHC as a single unit during division, thus generating one daughter cell that retains that ability to present antigen even after multiple cell divisions. This asymmetry impacts models of differentiation (Dustin and Meyer-Hermann, 2012; Thaunat and Batista, 2012). The TCR taken up by B cells are often in one structure that must contain many microvesicles. If this structure is inherited as a single quanta during the cell cycle, then the TCR-enriched microvesicles would also be asymmetrically partitioned, generating one daughter cell that would drop out of the cell cycle quickly. This could skew the counting process in an interesting way and would also have implications for B cell differentiation (Dustin and Meyer-Hermann, 2012; Thaunat and Batista, 2012).

### Immunologically Active Exosomes

Exosomes are small vesicles in the 60–100 nm range that are released from many cells types and which have biological activity when taken up by other cells (Johnstone et al., 1987; Thery, 2011). These particles have the same topology as the TCR-enriched microvesicles, with receptor ectodomains on the outside, and the ESCRT machinery generates both (Figure 1C). Exosomes from mast cells have been shown to have mitogenic activity in lymphocytes, although this has no demonstrated antigen specificity (Skokos et al., 2001). Exosomes generated by T cells that are activated by solid-phase anti-CD3 contain phosphorylated TCR components that are 2- to 3-fold enriched in comparison to exosomes generated in the steady state (Blanchard et al., 2002). They also contain CD40L (Blanchard et al., 2002). Studies in mast cells have also revealed that exosomes contain nucleic acids (Valadi et al., 2007). T cell exosomes also contain microRNAs that can influence expression of target genes in B cells (Mittelbrunn et al., 2011). The exosomes studies by Mittelbrunn et al. were generated by a distinct molecular mechanism compared to the plasma membrane-derived TCR-enriched microvesicles in that release of the exosomes required ESCRT-0 component HRS and sphingomyelinase activity, whereas neither is required for immunological synapse formation (Vardhana et al., 2010). However, it remains possible that both exosomes and TCR-enriched microvesicles will have a role in reprogramming antigen-presenting cells through antigen-specific delivery of highly stable microRNA complexes. The presence of CD40L in exosomes indicates that TCR-enriched exosomes or microvesicles may provide antigen-specific targeting of CD40 into the B cell. Endothelial cells can signal through CD40 after its endocytosis (Chen et al., 2006).

### Other Examples of Membrane Exchange

Immune cells have been documented to exchange membrane fragments or even to form membrane bridges since the early 1970s (Davis, 2007). Bona and colleagues described transfer lipopolysaccharide from macrophages to lymphocytes (probably B cells) as a prequel to antibody production (Bona et al., 1973). This example may reflect a specialized version of immune complex transfer that has been studied in more detail recently (Carrasco and Batista, 2007; Phan et al., 2009), although the form of the LPS was not determined. Transfer of antigenic MHC class I complexes from DC to CD4^+^ helper T cells has been shown to enhance T cell help for cytotoxic T cell generation (Ahmed et al., 2008). On the other hand, CD8 T cell capture of antigenic MHC class I complexes can lead to “fratricide,” which may have a role in limiting CTL responses. The direct acquisition of antigenic MHC class I complexes through a TCR-dependent process and then the potential representation of this complex leading to activation or killing is relatively straightforward. This is an example of “trogocytosis,” or gnawing of molecules off one cell by another (Joly and Hudrisier, 2003). It is unknown how much of trogocytosis is facilitated by ESCRT-mediated microvesicle generation. It is less obvious how a T with an MHC class II-restricted receptor could harvest MHC class I complexes from an antigen-presenting cell. One possibility is that MHC class I and II complexes have been shown to be coassociated in tetraspanin domains (Szöllósi et al., 1996), but since class I complexes are loaded in the endoplasmic reticulum and class II complexes are loaded in endosomes, it is not obvious how complexes with different peptides from the same antigen would be coassociated in the transferred membrane patches. Membrane bridges have also been observed between CTL and targeted cells, and such an event might allow for efficient diffusion of many highly mobile MHC class I complexes from target cells (Stinchcombe et al., 2001). In addition to antigen, BCR transfer has also been noted with bystander naive B cells having the capacity to acquire BCR for antigen activated B cells (Quah et al., 2008). No mechanism was determined, but the process took place at 4°C, which suggests some type of transient membrane fusion, perhaps nanotubes (Quah et al., 2008). While antigen enhanced, this mechanism did not appear to be suitable for antigen counting. The same group has also observed sharing of TCR between CD8 T cells in vivo (Quah et al., 2008). The TCR transfer is temperature independent and thus not the result of a classical immunological synapse, which is temperature dependent. The transfer is also not directly triggered by antigen, and it is proposed to facilitate the generation of many functional CTL to allow a more efficient and rapid response to pathogens, rather than an antigen-counting mechanism.

### Antigen-Specific Soluble Factors

The immunology literature from the late 1970s to the early 1980s was dominated by the study of soluble antigen-specific factors that could enhance or suppress immune responses (Guy et al., 1989; Kontiainen et al., 1978). While there were clear biological activities, this avenue of investigation was abandoned because the molecular characterization of the factors was genetically and biochemically intractable at the time. We now know that soluble TCR would not have sufficient affinity for MHC-peptide complexes to bind in an antigen-specific manner to pMHC on antigen-presenting cells at the concentrations reported for these factors (Davis et al., 1998). Therefore, soluble antigen-specific factors would almost certainly need to be multivalent. In fact, the mitogenic activity that I proposed above for TCR enriched microvesicles is very similar to the activity of helper factors described by the Hodes group in 1989 (Guy et al., 1989). The only problem with this potential connection is that the soluble antigen-specific factors were not removed by ultracentrifugation. Nonetheless, the smallest exosomes (30 nm) are similar in size to soluble lipoproteins, and it remains possible that the very specific conditions developed by investigators to generate these factors might have selected for vesicle populations that were on the borderline between soluble and particulate. Further study will be needed to determine if TCR-enriched microvescicles or endosomes can have immunosuppressive or stimulatory functions in vivo.

### Conclusions

Recent studies demonstrate that T cells have single-molecule sensitivity, and direct visualization of interactions currently supports a model in which T cell signaling in response to pMHC is a switch-like process. Therefore, the duration of signaling may be integrated, but counting may be challenging. B cells similarly utilize their antigen receptors to physically capture antigen and make pMHC complexes that allow the B cell to get CD154 and cytokines from T cells. How B cells would be programmed to respond in proportion to pMHC numbers they present is still not clear. This counting problem could be simplified if the B cell received a limited number of structures from T cells that would support division until lost by dilution. I would suggest that TCR-enriched microvesicles and related exosomes could provide such a counter. Other modes of membrane exchange exist in the immune system, but only the TCR-enriched microvesicles have the direct link to pMHC based on current data. This is a hypothesis based on in vitro data, and it will be critical to devise ways to test this in vivo. The generation of TCR-enriched microvesicles resembles retroviral budding in several respects, including the role of ESCRTs, and we have found that HIV can actually take over this process to generate virus-like particles in the immunological synapse (Choudhuri et al., 2014). Whether this basic mechanism evolved first in the immune system or was stolen back from viruses, the TCR-enriched microvesicles may function to convey self-limiting mitogenic signals to B cells that are proportional to pMHC. It will be intriguing in the future to determine if nucleic acids in the microvesicles and exosomes regulate the immune response (Mittelbrunn et al., 2011).
